# A Case of Small-Pox Recurring a Third Time after Vaccination, When It Proved Fatal

**Published:** 1851-10

**Authors:** 


*A Case of Small-Pox recurring a third time after Vaccination, when it proved fatal.*—Dr. John Webster communicated an example of this to the Royal Medical and Chirurgical Society, Feb. 26, 1851. After alluding to the fact that hooping-cough, measles, and scarlatina generally occur only once during the lifetime of an individual, exceptions, nevertheless, to the above rule, as well in these complaints as in small-pox, have been recorded byauthors. Three well-mar ed examples of the recurrence of small-pox met with in the same family are related, one of whic terminated fatally. The case especially referred to by Dr. Webster was that of H. N. N——, who had been vaccinated satisfactorily in 1827, when three months old. Notwithstanding this circumstance, he became attacked by small-pox in 1833, along with an elder brother, who had been likewise vaccinated. Both patients recovered, and nothing more was thought of the matter till 1838, when the two lads were again attacked by variola, along with another—that is a third—brother, likewise regularly vaccinated. However, all three got quite well in due time. Subsequently, Mr. H. N. N——, whose case is now just mentioned, went to India in the Company's service, where he was seized, in April last, with the usual and well-marked symptoms of small-pox, which soon became confluent, and proved fatal at Dharwarinth, on the 13th of that month: this making the third time this gentleman had been attacked by variola, although previously vaccinated.—*American Journ. Med. Sci.*

